# Supplementary material for: SLC35A5 Protein—A Golgi Complex Member with Putative Nucleotide Sugar Transport Activity
Source: Int J Mol Sci. 2019 Jan 11;20(2):276. doi: 10.3390/ijms20020276 (PMC6359379; doi:10.3390/ijms20020276)
Supplement: Supplementary file 1 [file ijms-20-00276-s001.pdf]

Supplementary materials content:

1. Cloning strategy

2. Supplementary figure 1 - Generation of SLC35A5 gene knock-out cells with details of KO confirmation

3. Supplementary figure 2 - Analysis of N- and O-glycans synthesized by wild type and SLC35A5-deficient HepG2 cells using lectins

4. Supplementary figure 3 - FACS analysis of the surface glycoconjugates of HepG2 wild type and SLC35A5-deficient cells

5. Supplementary figure 4 - N-glycan profiles of wild type and SLC35A5-deficient HepG2 cells

6. Supplementary figure 5 - Overexpression of SLC35A5 protein in MDCK-RCAr and CHO-Lec8 cells

7. Supplementary figure 6 - SLC35A5 amino-acid sequence

8. Supplementary table 1 - List of plasmids used in this study

9. Supplementary table 2 - List of lectins used in this study

10. Supplementary table 3 - List of antibodies used in this study

11. Supplementary table 4 - *In vivo* FLIM-FRET analysis of interactions between SLC35A5 protein and other members of SLC35A protein subfamily

CLONING STRATEGY

A. Cloning of human SLC35A5 to pSelect and pVitro expressing vectors

The ORF of SLC35A5 was cloned into pSelect-zeo and pVitro-neo plasmids (Invivogen) (in the case of pVitro MCS #1 was used), using BamHI and NheI restrictions sites. The forward primer contained additional Eco3I restriction site to avoid digestion of the SLC35A5 product by BamHI endonuclease. In the reverse primer, the sequence coding HA-tag or c-myc-tag was introduced.

Forward primer: HsA5Eco3_BamH_SE-F

cagt***ggtctcggatcc***ATGGAAAAACAGTGCTGTAG

Eco3I site is marked in yellow, BamHI in green, anealing sequence is underlined.

Reverse primer: HsA5Nhe_HA_R

ctag*gctagc*TTATGCGTAGTCTGGTACGTCGTATGGGTAGAAAGTATCTTCATCTGACTCATC

NheI site is marked in yellow, STOP codon in blue, anealing sequence is underlined, HA-tag sequence is marked in grey.

Reverse primer: HsA5Nhe_HA_R

ctag*gctagc*TTACAGATCTTCTTCAGAAATAAGTTTTTGTTCTGCGAAAGTATCTTCATCTGACTCATC

NheI site is marked in yellow, STOP codon in blue, anealing sequence is underlined, HA-tag sequence is marked in grey.

CAGATCTTCTTCAGAAATAAGTTTTTGTTCTGC

B. Cloning of human SLC35A5 to GFP vector

The same strategy like in [4] was used to insert SLC35A5 ORF into pTagGFP2-C vector (Evrogen)

C. Cloning of Mgat1 with c-myc tag to pSelect plasmid

The strategy was identical to those described in [4], with HA reverse sequence TGCGTAGTCTGGTACGTCGTATGGGTA (in the reverse primer used to amplify the Mgat-tag insert) replaced by c-myc reverse sequence CAGATCTTCTTCAGAAATAAGTTTTTGTTCTGC.

2

The open reading frame (ORF) of SLC35A5. Sequences used to design guide RNAs for knock-out (CRISPR-Cas) experiments (Santa Cruz Biotechnology) are marked in yellow. Control sequences for knock-out checking used in RT-PCR are marked in green.

ATGGAAAAACAGTGCTGTAGTCATCCTGTAATATGCTCCTTGTCAACAATGTATACATTCCTGCTAGGTGCCATA
TTCATTGCTTTAAGCTCAAGTCGCATCTTACTAGTGAAGTATTCTGCCAATGAAGAAAACAAGTATGATTATCTT
CCAACTACTGTGAATGTGTGCTCAGAACTGGTGAAGCTAGTTTTCTGTGTGCTTGTGTCATTCTGTGTTATAAAG
AAAGATCATCAAAGTAGAAATTTGAAATATGCTTCCTGGAAGGAATTCTCTGATTTCATGAAGTGGTCCATTCCT
GCCTTTCTTTATTTCCTGGATAACTTGATTGTCTTCTATGTCCTGTCCTATCTTCAACCAGCCATGGCTGTTATC
TTCTCAAATTTTAGCATTATAACAACAGCTCTTCTATTCAGGATAGTGCTGAAGAGGCGTCTAAACTGGATCCAG
TGGGCTTCCCTCCTGACTTTATTTTTGTCTATTGTGGCCTTGACTGCCGGGACTAAAACTTTACAGCACAACTTG
GCAGGACGTGGATTTCATCACGATGCCTTTTTCAGCCCTTCCAATTCCTGCCTTCTTTTCAGAAGTGAGTGTCCC
AGAAAAGACAATTGTACAGCAAAGGAATGGACTTTTCCTGAAGCTAAATGGAACACCACAGCCAGAGTTTTCAGT
CACATCCGTCTTGGCATGGGCCATGTTCTTATTATAGTCCAGTGTTTTATTTCTTCAATGGCTAATATCTATAAT
GAAAAGATACTGAAGGAAGGGAACCAGCTCACTGAAAGCATCTTCATACAGAACAGCAAACTCTATTTCTTTGGC
ATTCTGTTTAATGGGCTGACTCTGGGCCTTCAGAGGAGTAACCGTGATCAGATTAAGAACTGTGGATTTTTTTAT
GGCCACAGTGCATTTTCAGTAGCCCTTATTTTTGTAACTGCATTCCAGGGCCTTTCAGTGGCTTTCATTCTGAAG
TTCCTGGATAACATGTTCCATGTCTTGATGGCCCAGGTTACCACTGTCATTATCACAACAGTGTCTGTCCTGGTC
TTTGACTTCAGGCCCTCCCTGGAATTTTTCTTGGAAGCCCCATCAGTCCTTCTCTCTATATTTATTTATAATGCC
AGCAAGCCTCAAGTTCCGGAATACGCACCTAGGCAAGAAAGGATCCGAGATCTAAGTGGCAATCTTTGGGAGCGT
TCCAGTGGGGATGGAGAAGAACTAGAAAGACTTACCAAACCCAAGAGTGATGAGTCAGATGAAGATACTTTCTAA

Human SLC35A5 sequence containing HA-tag at C-terminus (yellow)

MEKQCCSHPVICSLSTMYTFLLGAIFIALSSSRILLVKYSANEENKYDYLPTTVNVCSELVKLVFCVLVSFCVIK
KDHQSRNLKYASWKEFSDFMKWSIPAFLYFLDNLIVFYVLSYLQPAMAVIFSNFSIITTALLFRIVLKRRLNWIQ
WASLLTLFLSIVALTAGTKTLQHNLAGRGFHHDAFFSPSNSCLLFRSECPRKDNCTAKEWTFPEAKWNTTARVFS
HIRLGMGHVLIIVQCFISSMANIYNEKILKEGNQLTESIFIQNSKLYFFGILFNGLTLGLQRSNRDQIKNCGFFY
GHSAFSVALIFVTAFQGLSVAFILKFLDNMFHVLMAQVTTVIITTVSVLVFDFRPSLEFFLEAPSVLLSIFIYNA
SKPQVPEYAPRQERIRDLSGNLWERSSGDGEELERLTKPKSDESDEDTFYPYDVPDYA

Human SLC35A5 sequence containing c-myc-tag at C-terminus (yellow)

MEKQCCSHPVICSLSTMYTFLLGAIFIALSSSRILLVKYSANEENKYDYLPTTVNVCSELVKLVFCVLVSFCVIK
KDHQSRNLKYASWKEFSDFMKWSIPAFLYFLDNLIVFYVLSYLQPAMAVIFSNFSIITTALLFRIVLKRRLNWIQ
WASLLTLFLSIVALTAGTKTLQHNLAGRGFHHDAFFSPSNSCLLFRSECPRKDNCTAKEWTFPEAKWNTTARVFS
HIRLGMGHVLIIVQCFISSMANIYNEKILKEGNQLTESIFIQNSKLYFFGILFNGLTLGLQRSNRDQIKNCGFFY
GHSAFSVALIFVTAFQGLSVAFILKFLDNMFHVLMAQVTTVIITTVSVLVFDFRPSLEFFLEAPSVLLSIFIYNA
SKPQVPEYAPRQERIRDLSGNLWERSSGDGEELERLTKPKSDESDEDTFAEQKLISEEDL

3

SUPPLEMENTARY FIGURE 1 Generation of *SLC35A5* gene knock-out cells. Genomic DNA and RNA were isolated from HepG2 wild type cells and several stable transfectants and either PCR (DNA) or RT-PCR (mRNA) reaction was performed using *SLC35A5* gene-specific primers. Products were separated in 2.5 % (w/v) agarose gel and visualized with ethidium bromide.

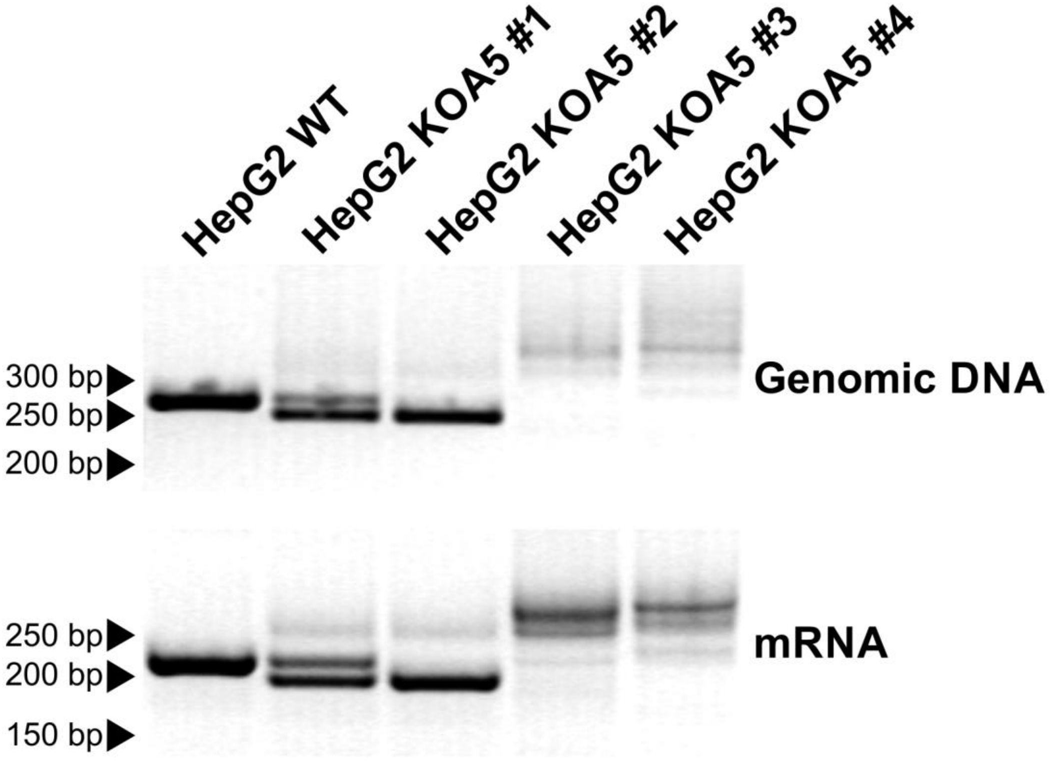

SEQUENCING OF FRAGMENTS AMPLIFIED ON GENOMIC DNA ISOLATED FROM HEPG2 WILD TYPE AND HEPG2 SLC35A5-KO (CLONE #2) CELLS

```
      1                                                                     70
 wt1  ATGGAAAAAC AGTGCTGTAG TCATCCTGTA ATATGCTCCT TGTCAACAAT GTATACATTC CTGCTAGGTG
KOA5  ATGGAAAAAC AGTGCTGTAG TCATCCTGTA ATATGCTCCT TGTCAACAAT GTATACATTC CTGCT-----
      71                                                                   140
 wt1  CCATATTCAT TGCTTTAAGC TCAAGTCGCA TCTTACTAGT GAAGTATTCT GCCAATGAAG GTAAGTTAAG
KOA5  ---------- ---------- -CAAGTCGCA TCTTACTAGT GAAGTATTCT GCCAATGAAG GTAAGTTAAG
      141                                                                  210
 wt1  ACTTGGTATA TGCATGGAGC ACTTCCATCT AATCACACAT CTCTCTCTTG CCTTTGGTTC TGTTATATAT
KOA5  ACTTGGTATA TGCATGGAGC ACTTCCATCT AATCACACAT CTCTCTCTTG CCTTTGGTTC TGTTATATAT
      211                                                                  280
 wt1  AACATGGAAA TAATAATGCC TTTTGCTTCA TGTGAGTGAT AAAGCATATT TAAATTTGAT TATTTAACCT
KOA5  AACATGGAAA TAATAATGCC TTTTGCTTCA TGTGAGTGAT AAAGCATATT TAAATTTGAT TATTTAACCT

      281
 wt1  TGCATTCCTC AACAAGA
KOA5  TGCATTCCTC AACAAGA
```

Exon #1 marked in red/yellow, intron #1 printed in blue, 26-bp deletion (---------) is shown in KO-genomic DNA

Potential translation of Slc35A5-KO DNA:
MEKQCCSHPVICSLSTMYTFLLKSHLTSEVFCQ- (STOP codon at pos. 34)

4

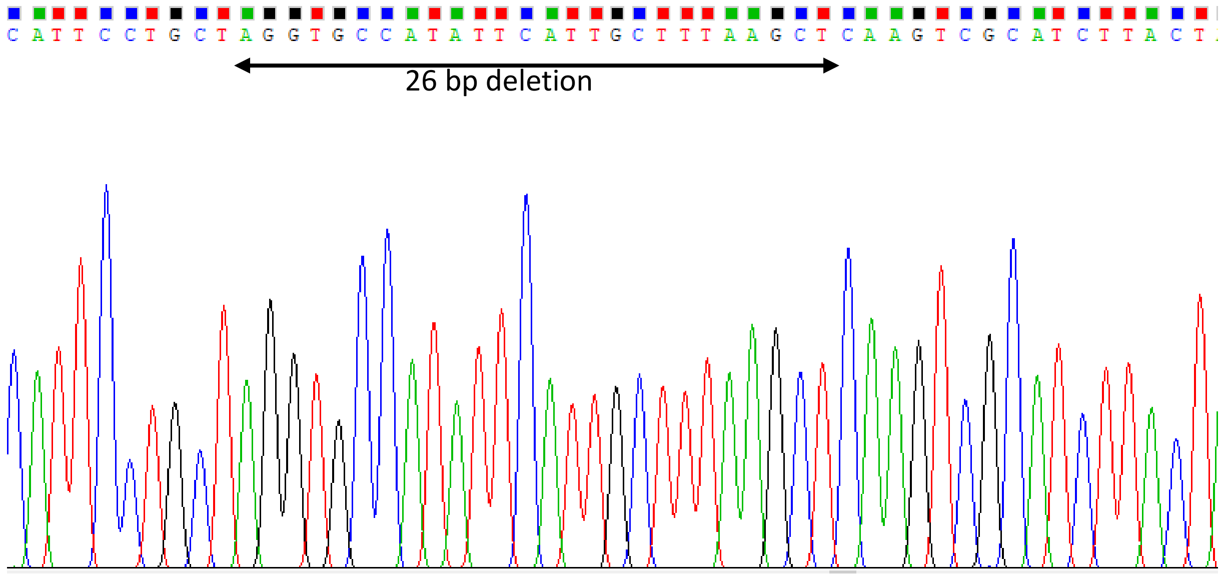

Wild type genomic DNA (deletion region)

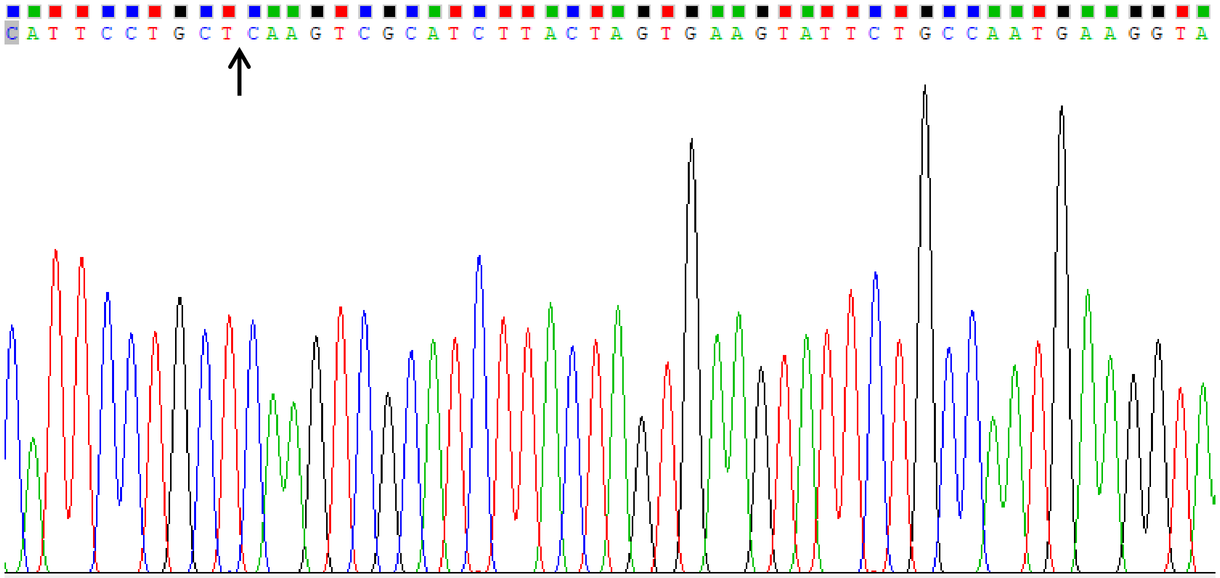

SLC35A5-KO genomic DNA (deletion region), repair ligation site is pointed

DNA fragments amplified on genomic DNA and cloned into pJet1.2 sequencing vector

Genomic DNA cloned into pJet1.2 sequencing vector	Total pJet1.2 sequenced clones	WT clones	Clones with 26-bp deletion	% of wt clones
HepG2 WT cells	10	10	0	100
Clone #1	10	2	8	25
Clone #2	10	0	10	0
Clone #3	no PCR product	NA	NA	NA
Clone #4	no PCR product	NA	NA	NA

5

SUPPLEMENTARY FIGURE 2 Analysis of *N*- and *O*-glycans synthesized by wild type and SLC35A5-deficient HepG2 cells using lectins. *N*- and *O*-glycans were detected after SDS-PAGE separation of the cell lysates using specific lectins. Lectins specificity is listed in supplementary table 2S.

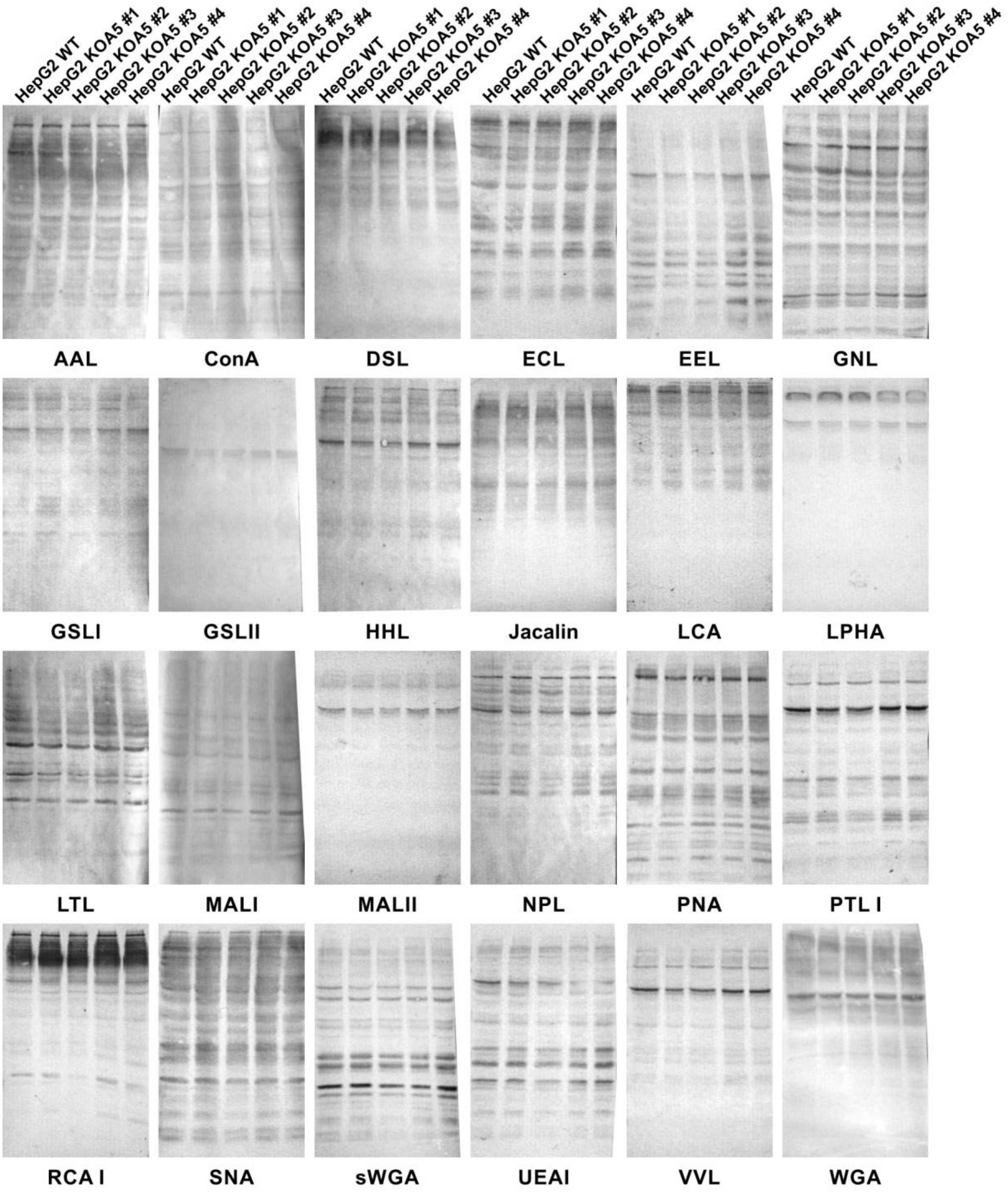

6

SUPPLEMENTARY FIGURE 3 FACS analysis of the surface glycoconjugates of HepG2 wild type and SLC35A5-deficient cells. Seven different lectins were used to analyze HepG2 wild-type (black) and *SLC35A5* knock-out (grey) cells. The data are presented as a mean fluorescence intensity of two independent biological replicates ± SEM. Lectins specificity is listed in supplementary table 2S.

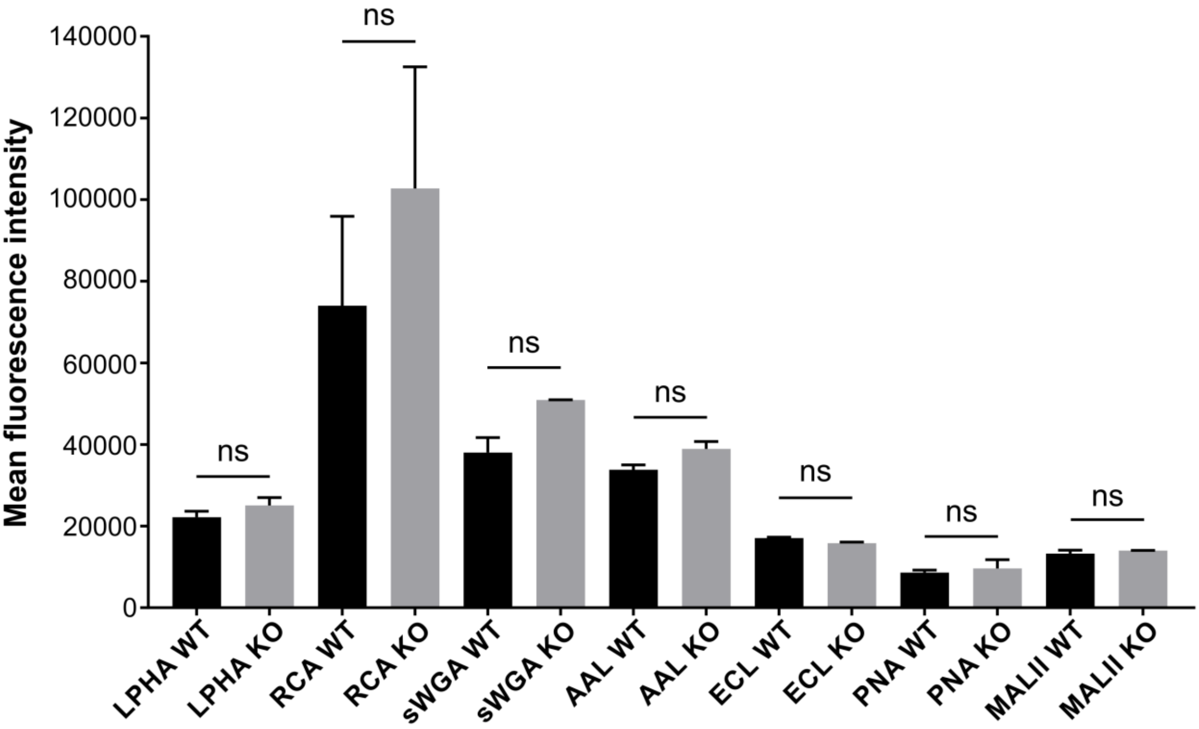

SUPPLEMENTARY FIGURE 4 *N*-glycan profiles of wild type and SLC35A5-deficient HepG2 cells. *N*-glycans were enzymatically released from glycoproteins produced by wild type and SLC35A5-deficient HepG2 cells, fluorescently labeled with 2-AB, purified and separated on the GlycoSep N column using HPLC. Representative data from three independent separations with a similar tendency for wild type cells and all clones are shown.

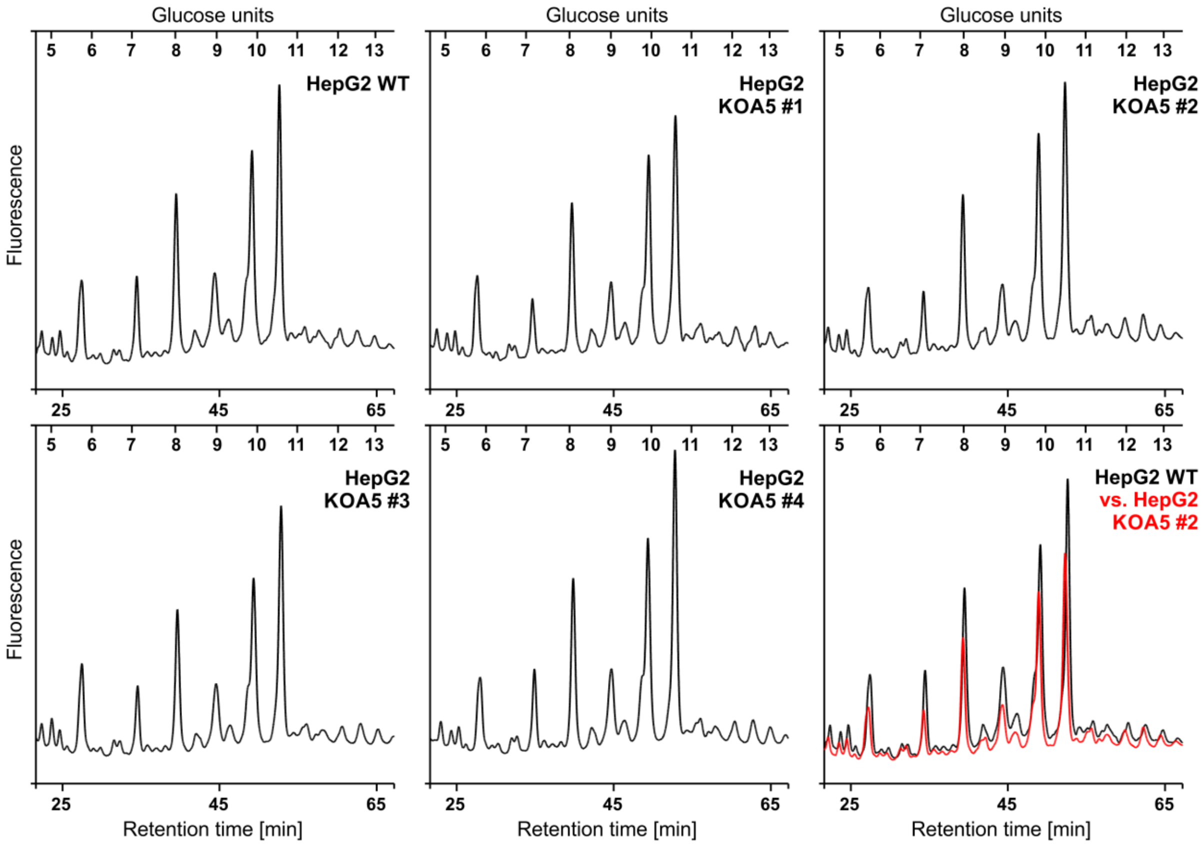

8

SUPPLEMENTARY FIGURE 5 Overexpression of SLC35A5 protein in MDCK-RCA^r and CHO-Lec8 cells. Cell lysates were subjected to SDS-PAGE and Western blotting with anti-HA-HRP antibody.

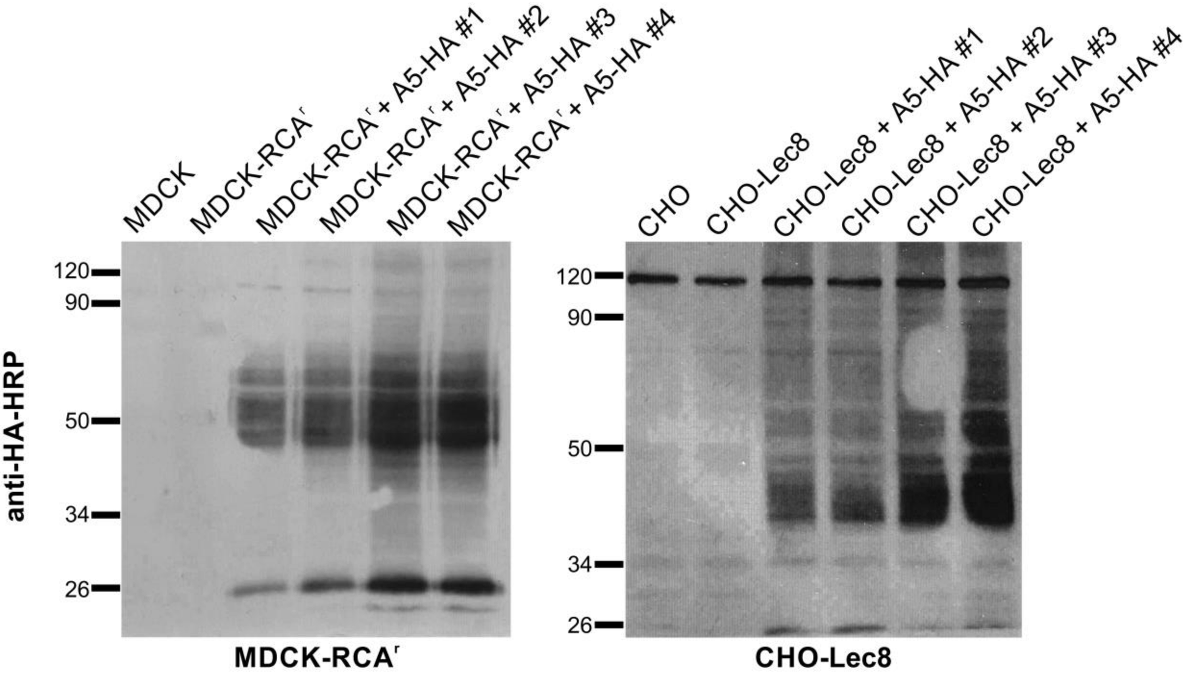

9

SUPPLEMENTARY FIGURE 6 SLC35A5 amino acid sequence. DXEE, DXXD and DXD motifs are indicated with black boxes.

```
1    MEKQCCSHPVICSLSTMYTFLLGAIFIALSSSRILLVKYSANEENKYDYL   50
51   PTTVNVCSELVKLVFCVLVSFCVIKKDHQSRNLKYASWKEFSDFMKWSIP   100
101  AFLYFLDNLIVFYVLSYLQPAMAVIFSNFSIITTALLFRIVLKRRLNWIQ   150
151  WASLLTLFLSIVALTAGTKTLQHNLAGRGFHHDAFFSPSNSCLLFRSECP   200
201  RKDNCTAKEWTFPEAKWNTTARVFSHIRLGMGHVLIIVQCFISSMANIYN   250
251  EKILKEGNQLTESIFIQNSKLYFFGILFNGLTLGLQRSNRDQIKNCGFFY   300
301  GHSAFSVALIFVTAFQGLSVAFILKFLDNMFHVLMAQVTTVIITTVSVLV   350
351  FDFRPSLEFFLEAPSVLLSIFIYNASKPQVPEYAPRQERIRDLSGNLWER   400
401  SSGDGEELERLTKPKSDESDEDTF                             424
```


10

SUPPLEMENTARY TABLE 1 List of plasmids used in this study.

Plasmid name	Original vector	Insert	Reference
pTag-GFP2-C-A5	pTag-GFP2-C	SLC35A5	This study
pTag-RFP-C-A1	pTag-RFP-C	SLC35A1	[4]
pTag-RFP-C-A2-ER	pTag-RFP-C	SLC35A2-ER	[40]
pTag-RFP-C-A2-Golgi	pTag-RFP-C	SLC35A2-Golgi	[20]
pSelect-RFP-A3	pSelect	RFP- SLC35A3	[40]
pTag-RFP-C-A4	pTag-RFP-C	SLC35A4	[4]
pTag-RFP-C-A5	pTag-RFP-C	SLC35A5	[4]
pVitro-A5-HA	pVitro	SLC35A5-HA	This study
pSelect-A5-HA	pSelect	SLC35A5-HA	This study
pSelect-Mgat1-c-myc	pSelect	Mgat1-c-myc	This study
pSelect-A5-c-myc	pSelect	SLC35A5-c-myc	This study
pSelect-Mgat1-HA	pSelect	Mgat1-HA	[4]

11

SUPPLEMENTARY TABLE 2 List of lectins used in this study.

Lectin	Full name	Specificity	Dilution
AAL	*Aleuria aurantia* lectin	fucose (α-1,6) *N*-acetylgalactosamine or fucose (α -1,3) *N*-acetyllactosamine	1:1000
ConA	*Canavalia ensiformis* agglutinin	α-linked mannose, glucose	1:1000
DSL	*Datura stramonium* lectin	*N*-acetylglucosamine	1:1000
ECL	*Erythrina cristagalli* lectin	galactose, *N*-acetylgalactosamine, lactose	1:1000
EEL	*Euonymus europaeus* lectin	galactosyl (α-1,3) galactose	1:250
GNL	*Galanthus nivalis* lectin	(α-1,3) mannose	1:250
GSL I	*Griffonia simplicifolia* lectin I	α-*N*-acetylgalactosamine, α-galactose	1:500
GSL II	*Griffonia simplicifolia* lectin II	α- or β-linked *N*-acetylglucosamine	1:250
HHL	*Hippeastrum hybrid* lectin	α-linked mannose	1:250
Jacalin	-	galactose in *O*-glycans	1:1000
LCA	*Lens culinaris* agglutinin	α-linked mannose, glucose	1:1000
L-PHA	*Phaseolus vulgaris* leucoagglutinin	galactose in complex *N*-glycans	1:250
LTL	*Lotus tetragonolobus lectin*	α-linked fucose	1:500
MAL I	*Maackia amurensis* lectin I	galactose (β-1,4) *N*-acetylgalactosamine	1:500
MAL II	*Maackia amurensis* lectin II	(α-2,3) sialic acid	1:250
NPL	*Narcissus pseudonarcissus* lectin	α-linked mannose	1:500
PNA	*Arachis hypogaea* agglutinin	galactosyl (β-1,3) *N*-acetylgalactosamine	1:500
PTL I	*Psophocarpus tetragonolobus* lectin I	α-linked *N*-acetylgalactosamine	1:500
RCA I	*Ricinus communis* agglutinin I	galactose, lactose	1:1000
SNA	*Sambucus nigra* lectin	(α-2,6) sialic acid	1:1000
sWGA	*Wheat germ* agglutinin	*N*-acetylglucosamine and sialic acid	1:500
UEA I	*Ulex europaeus* agglutinin	(α-1,2) fucose	1:500
VVL	*Vicia villosa* lectin	α- or β-linked terminal *N*-acetylgalactosamine	1:250
WGA	succinylated *Wheat germ* agglutinin	*N*-acetylglucosamine	1:1000

12

SUPPLEMENTARY TABLE 3 List of antibodies used in this study. IF – immunofluorescence; WB – Western blotting.

Antibody	Clonality	Dilution IF	Dilution WB	Host	Company
anti-HA	Polyclonal	1:500	1:5000	Rabbit	Abcam
anti-c-myc	Polyclonal	1:1000	-	Chicken	Abcam
anti-calnexin	Polyclonal	1:100	1:2500	Rabbit	Abcam
anti-syntaxin 16	Monoclonal	1:500	1:2500	Rabbit	Abcam
anti-β-tubulin	Monoclonal	1:200	-	Mouse	Sigma Aldrich
anti-HA-Alexa Fluor 647	Monoclonal	1:100	-	Mouse	BioLegend
anti-mouse Alexa Fluor 633	Polyclonal	1:200	-	Goat	Molecular Probes
anti-rabbit Alexa Fluor 555	Polyclonal	1:200	-	Goat	Molecular Probes
anti-chicken Alexa Fluor 488	Polyclonal	1:200	-	Goat	Molecular Probes
anti-SLC35A2	Polyclonal	-	1:1000	Rabbit	Abcam
anti-GM130	Monoclonal	-	1:1000	Mouse	BD Biosciences
anti-Mgat1	Polyclonal	-	1:1000	Rabbit	Abcam
anti-keratan sulfate (MAB2022)	Monoclonal	-	1:5000	Mouse	Merck Millipore
anti-chondroitin-4-sulfate (MAB2030)	Monoclonal	-	1:5000	Mouse	Merck Millipore
anti-chondroitin sulfate A (2H6)	Monoclonal	-	1:1000	Mouse	AMS Biotechnology
anti-heparan sulfate (F69-3G10)	Monoclonal	-	1:1000	Mouse	AMS Biotechnology
anti-HA-HRP	Monoclonal	-	1:500	Rat	Sigma Aldrich
anti-mouse HRP	Polyclonal	-	1:10000	Goat	Promega
anti-rabbit HRP	Polyclonal	-	1:10000	Goat	Sigma Aldrich

13

SUPPLEMENTARY TABLE 4 *In vivo* FLIM-FRET analysis of interactions between SLC35A5 protein and other members of SLC35A protein subfamily.

FRET combination	n	$\tau_{average}$ (ns)	τ_{short} (ns)	τ_{long} (ns)	χ^2
A5-eGFP alone	19	2.70±0.04			1.02±0.06
A5-eGFP + mRFP-A1	29	2.29±0.14	1.15±0.32	2.70	0.99±0.06
A5-eGFP + mRFP-A2-Golgi	34	2.24±0.12	1.35±0.13	2.70	0.98±0.06
A5-eGFP + mRFP-A2-ER	29	2.31±0.20	1.20±0.38	2.70	1.02±0.08
A5-eGFP + mRFP-A3	34	2.35±0.09	1.54±0.09	2.70	1.03±0.07
A5-eGFP + mRFP-A4	32	2.39±0.12	1.20±0.21	2.70	1.01±0.06
A5-eGFP + A5-mRFP	38	2.51±0.09	1.15±0.34	2.70	1.00±0.05

14
